# The moderating role of job satisfaction in the relationship between workplace violence and depression among accountants

**DOI:** 10.3389/fpubh.2026.1816538

**Published:** 2026-05-29

**Authors:** Tugçe Uzun Kocamiş, Gülçin Kazan

**Affiliations:** 1 Vocational School of Social Sciences, Istanbul University Cerrahpaşa, Istanbul, Türkiye; 2 Faculty of Business and Management Sciences, Istanbul Sabahattin Zaim University, Istanbul, Türkiye

**Keywords:** accountants, depression, employee wellbeing, job satisfaction, moderation analysis, occupational health, workplace violence

## Abstract

**Background:**

Workplace violence is a significant issue within organizations that relates to psychological wellbeing and work performance. Numerous studies have shown that workplace violence is related to adverse mental health consequences, especially depression. Using Social Exchange and Stress-Strain Theory perspectives, this paper explores the impact of negative workplace stressors on mental health impairment and how this relationship may be moderated by job satisfaction. Despite abundant literature on the topic, little research has been conducted on the highly stressful profession of accounting. This study seeks to examine the moderating role of job satisfaction on the association between workplace violence and depression amongst accountants.

**Method:**

A time-lagged multi-wave survey research design with a quantitative approach was used for this study. A total of 325 participants, all accountants from different organizations, were included in the study and surveyed across three consecutive waves, each separated by a 30-day interval. Scales measuring workplace violence, job satisfaction, and depression were utilized for data collection.

**Results:**

A positive correlation existed between workplace violence and depression (β = 0.360, *p* < 0.001) whereas a negative correlation existed between workplace violence and job satisfaction (β = −0.311, *p* < 0.001). The moderating effect of job satisfaction was significant (β = −0.245, *p* < 0.001), indicating that higher levels of job satisfaction weaken the relationship between workplace violence and depression.

**Conclusion:**

The findings highlight the role of job satisfaction in the relationship between workplace violence and employee wellbeing, particularly in high-stress occupations such as accounting. The results also provide implications for organizational interventions aimed at enhancing job satisfaction among employees.

## 1 Introduction

Workplace violence is negatively associated with employees' mental health and corporate performance (1). It is associated with higher levels of stress, anxiety, and depression, which are in turn linked to lower willingness to work and reduced productivity. Consequently, the turnover rates are likely to be higher. This has been supported through the theoretical model suggested by (2, 3). In particular, work participation becomes weaker in depressed employees and therefore the employee's participation in decision-making processes gets disrupted (4). In this process, an important factor associated with employees' intention to stay at work, their mental health, and the potential alleviation of such negativities is job satisfaction (5, 6). While workplace violence generally weakens employees' perception of justice, trust, and safety (7), high job satisfaction may help employees maintain their psychological state and is associated with lower negative effects (6).

Accounting represents a demanding occupational environment where complex intellectual tasks must be undertaken in combination with poor social relationships, heavy regulation, and rigorous workloads. For example, many accountants experience significant pressures to complete important reports on time, maintain proper financial accuracy, and uphold certain ethical standards in the workplace (8–14). Compared to many other professional fields, workplace violence among accountants typically takes non-physical forms, including verbal abuse, bullying, and psychological intimidation.

Nevertheless, despite its obvious applicability, there is a lack of attention in the research on workplace violence from the accounting profession, which has been concentrated primarily on the areas of public services and caregiving (7).

The most recent findings in the accounting field confirm the above context, as the relationship between job burnout and job stress and the wellbeing of accountants has been demonstrated. The empirical evidence shows that the impact of burnout is negative on psychological wellbeing and job satisfaction, whereas the latter is an essential mediating variable connecting the former to employee performance and turnover (8–10). Furthermore, job stress is regarded as a substantial determinant of job satisfaction and engagement within the accounting environment (11).

Apart from addressing the knowledge gap within the literature, the examination of workplace violence among accountants will add a new dimension to the conceptualization of how professionals with high responsibility levels perceive and process organizational abuse. As such, this approach forms the foundation for the understanding of psychosocial risks in the accounting industry, making this study one of the pioneers in this area.

Job satisfaction may play a unique role as a moderator within this industry. The constant exposure to workload pressure and ethical challenges may be offset by job satisfaction derived from fairness, positive supervision, and the significance of the job. Thus, this study aims to explore the role of job satisfaction as a moderator between workplace violence and depression within the accounting industry.

This research focuses on the relationship between workplace violence, job satisfaction, and depression within the accounting industry and, more specifically, whether job satisfaction can act as a moderator that reduces the impact of workplace violence on depression. In this aspect, the study aims to address this gap that exists in the literature. The findings shall add to informing business and policy makers in the design of strategies to enhance employees' wellbeing and reduce workplace violence.

## 2 Conceptual framework and literature review

### 2.1 Workplace violence and depression

Workplace violence is considered a very critical threat in workplaces, having severe impacts on employees' mental health. The Occupational Safety and Health Administration (15) explains workplace violence as “physical assaults, harassment, intimidation, or other threatening or disruptive behavior.” Workplace violence has also been shown to impact the mental health of individuals those exposed to it, exposing them to stress, anxiety, depression, and post-traumatic stress disorder (PTSD) (16). Workplace violence is significantly associated with depression because it chronically exposes staff to stress, emotional trauma, and psychological instability. Studies show that exposure to workplace violence is significantly associated with a higher risk of depression and is also linked to chronic stress (1, 2, 17). Moreover, high levels of exposure to workplace violence relate to burnout and emotional exhaustion and are often associated with disengagement from work, decreased motivation, and productivity (18). At this stage, research has highlighted the significance of organizational climate in relation to workplace violence. These studies suggest that employees, particularly in organizations with a strong organizational climate, may experience fewer negative outcomes (3, 19, 20). While organizational policies aimed at addressing workplace violence are considered important for employees' mental health, some workplace environments are also associated with varying levels of depression risk. In this regard, studies conducted in various sectors have established that work environments are associated with anxiety, stress, and other psychological repercussions (21–27). Though these studies emphasize organizational intervention strategies, Lagabrielle et al. (28) stressed that workplace violence is much more common in large enterprises than in small enterprises. This suggests that workplace violence may lead to more serious psychological consequences in small and hierarchical workplaces such as accounting firms (22, 26). Workplace violence has become an important research topic in the field of workplace psychology due to its direct effects on the mental health of employees. Indeed, studies have established a strong link between workplace violence and depression and burnout (29–33).

### 2.2 The moderating role of job satisfaction

Job satisfaction reflects employees' general feeling about their job and depends on many elements of a job: physical environment, earnings, job security, and interaction with other staff members or the opportunity for professional growth (34, 35). Job satisfaction is one of the most important issues in both individual and organizational perspectives. Satisfied employees have lower turnover rates (34, 36) and higher organizational commitment to work, and have lower levels of stress, anxiety, and depression as related to mental health (5, 37). However, strategies to increase job satisfaction include offering career development opportunities, creating a supportive work environment, and providing fair compensation.

Workplace violence may negatively affect the wellbeing and notably decrease job satisfaction among employees. The presence of workplace violence is associated with increased stress and anxiety among employees (17). Eventually, this situation is associated with lower job satisfaction (7). Continuous exposure to workplace violence is also associated with burnout and emotional exhaustion. These conditions are, in turn, associated with lower job satisfaction and morale (38). While workplace violence is associated with higher levels of depression, other studies suggest that job satisfaction may be associated with weaker negative effects (39–42). Moreover, job satisfaction may weaken the relationship between workplace violence and depression, as suggested by research on workplace conditions in conjunction with institutional interventions (43–47).

### 2.3 Theoretical model

This study investigates workplace violence in relation to job satisfaction and depression, based on both the stress-strain theory and social exchange theory. Put together, these frameworks provide a sound basis for understanding how workplace violence may affect the mental wellbeing of employees and how job satisfaction could help moderate that impact.

#### 2.3.1 Stress-strain theory

The stress-strain concept is a conceptual framework that deals with the physiological and psychological tension created by the disruption of balance between the environmental demands-workload, role ambiguity, conflicts, etc.-which individuals are confronted with, and their internal resources. This theory by Lazarus and Folkman (48) explains the negative effects of workplace stressors on the psychological health of individuals (49). This theory assumes that high job demands with low autonomy conditions raise employees' levels of stress, which, in turn, contribute to mental health problems (50). Failure to control that stress in the long term may have serious consequences for individuals, ranging from physical health problems to emotional exhaustion (4, 51).

Work stressors such as violence and harassment exceed the coping capacities of workers and are associated with psychological problems such as burnout and depression (52). In this context, job satisfaction is a significant factor. Those employees who have high job satisfaction-especially due to supportive leadership and effective conflict resolution mechanisms at the organizational level, which also apply employee support programs- may be better able to cope with stress factors and maintain their mental health.

According to the Stress–Strain Theory (49), exposure to workplace violence triggers the cognitive appraisals of threat and perceived injustice. These appraisals lead to negative emotional arousal and prolonged strain responses that, over time, eventuate as symptoms of depression. Thus, this mechanism provides the theoretical basis for predicting a positive association between workplace violence and depression.

In this perspective, Stress-Strain Theory emphasizes that although understanding workplace violence is crucial, organizational interventions play an important role in minimizing the level of stress through supporting the employees' psychological health (53).

#### 2.3.2 Social exchange theory

Social exchange theory is a theoretical model that focuses on social relations by means of the balance of reward and cost (54, 55). In such a point of view, individuals attach importance to each other's interest and benefit in interactions; such social relations serve to make a strong impact on workplace conduct and worker wellbeing (56).

This theory suggests that employees establish social exchange relationships with the employer, managers, and coworkers and create expectations that they will receive fair, trustworthy, and supportive treatment from these relationships (55). However, violence at work may destroy such a mutual expectation and affect job satisfaction, trust, and psychological wellbeing negatively (20). In more detail, violence could make employees feel useless and helpless, leading to lower job satisfaction and work motivation (7).

A lack of social exchange expectations can be associated with the loss of employee confidence, alienation, and job dissatisfaction, and can also open the door to mental disorders such as depression and anxiety (57). More supportive employees are exposed to fewer psychological effects of work violence and develop better coping strategies against such adversities (58–60).

According to Social Exchange Theory (58), a relationship of reciprocity between workers and their organizations exists. Violation of the psychological contract, therefore, might occur with workplace violence and result in lowering of trust and emotional withdrawal. On the other hand, high job satisfaction reflects a sense of organizational support and reciprocal balance, which shields against the negative emotional aftermath of violations.

The Stress–Strain Theory suggests that severe stressors such as workplace violence may amplify employees' cognitive threat appraisal, which, over time, is associated with sustained strain and depressive symptoms (48, 49). By contrast, Social Exchange Theory suggests that violation of the reciprocal relationship and psychological contract between employees and their organization undermines perceptions of trust and support and activates emotional withdrawal (54, 55). The job satisfaction variable figures at the juncture of these two theories: it operates both as a “psychological resource” in enabling employees to cope with stress (52) and as a concrete proxy of perceived organizational support (5, 37). Hence, high job satisfaction simultaneously is associated with lower strain reactions, as explained by Stress–Strain Theory, and attenuates the perception of psychological contract violation, as described by Social Exchange Theory.

Theoretically, job satisfaction can play the moderating role between workplace violence and depression based on both the Stress–Strain and Social Exchange Theories. From the perspective of the Stress–Strain Theory (27, 48), workplace violence is a kind of chronic stressor that breaks the balance between job demands and coping resources, thus leading to emotional exhaustion and depressive symptoms. Job satisfaction may play a role in individuals' internal coping mechanisms and is associated with higher self-efficacy and perceived control, as well as lower levels of stress.

In line with Social Exchange Theory (55, 56), workplace relationships are based on fairness, trust, and reciprocity. Violence at work violates this psychological contract and fosters emotional withdrawal, whereas high job satisfaction reflects organizational support and restores relational balance. Integrating these perspectives, job satisfaction serves as both a psychological and relational resource—reducing strain and reinforcing fairness and belonging. This framework explains how job satisfaction moderates the impact of workplace violence on depression, especially in demanding professions such as accounting.

#### 2.3.3 Theoretical contribution

This study contributes to the literature by developing an integrated framework that combines stress-based and relational perspectives to explain the association between workplace violence and employee wellbeing. Rather than treating these perspectives separately, the study suggests how they jointly account for both the psychological and organizational dimensions of employees' responses to adverse workplace conditions.

A key contribution lies in reconceptualizing job satisfaction as a contextual resource rather than merely an outcome. In this framework, job satisfaction reflects both an individual coping capacity and a signal of perceived organizational support, thereby shaping how workplace stressors are experienced. This dual role provides a more nuanced understanding of how employee wellbeing is influenced by both internal and relational mechanisms.

In addition, the study extends existing knowledge by applying this integrated perspective to the accounting profession, a context that remains underexplored in workplace violence research. The findings suggest that workplace violence in this setting is often embedded in less visible, psychologically driven dynamics linked to hierarchical structures and performance pressures. This highlights the importance of considering occupational context as a boundary condition in understanding psychosocial risks.

Overall, the study contributes by offering a more context-sensitive and theoretically integrated explanation of the relationship between workplace violence and employee wellbeing.

## 3 Method

### 3.1 Research design

This study employs a time-lagged multi-wave survey design to examine the moderating role of job satisfaction in the relationship between workplace violence and depression among accountants. The research follows a quantitative methodology, utilizing a survey approach to collect data. Data were collected in three waves with 30-day intervals in order to reduce common method bias and ensure temporal separation between variables. The study's theoretical framework is based on Stress-Strain Theory and Social Exchange Theory, which provide insights into workplace violence's association with employees' mental wellbeing and the moderating role of job satisfaction.

To test the proposed Relationships and Examine the workplace influence violence and job satisfaction on depression following hypotheses have been formulated:

H1: Workplace violence against accountants is positively linked to their level of depression.

Drawing from Stress–Strain Theory, workplace violence represents chronic stressors elevating emotional strain and eventually depressive symptoms. Repeated experiences of verbal or physical aggression can break down employees' sense of fairness and personal control, which are critical antecedents of depression. Thus, we hypothesize that workplace violence is positively associated with depression.

H2: There is a negative relationship between accountants' depression level and their job satisfaction.

From a stress–strain perspective, job satisfaction represents a psychological resource that may help buffer the association between workplace stressors and negative outcomes. Higher levels of job satisfaction can be seen to indicate positive working conditions that may reduce emotional strain and shield a person from depressive symptoms.

H3: Job satisfaction moderates the relationship between workplace violence and the level of depression among accountants.

Drawing on Social Exchange Theory, job satisfaction is seen to reflect perceptions of organizational support and access to socio-emotional resources. Workplace violence may be perceived as a violation of the psychological contract, which in turn may diminish trust and lead to emotional withdrawal. However, high job satisfaction may reflect perceptions that the organization values and supports the employee and may be associated with resources that weaken the strain process described in Stress–Strain Theory. Consequently, we expect that the positive relationship between workplace violence and depression will be weaker for employees who report high levels of job satisfaction.

### 3.2 Sampling and precedent

This study employs a time-lagged multi-wave survey design. Data were collected from accountants employed in small and medium-sized enterprises (SMEs) as well as corporate firms across Türkiye. The data were collected through an online questionnaire distributed via Google Forms. The data collection process took place over a period of 7 months, from May to November 2024.

To mitigate the issue of common method bias (CMB), the survey was administered in three waves with 30-day intervals between each wave, ensuring temporal separation among the variables.

In addition to this, Harman's single factor test was used against the CMB problem. In this test, if a single factor emerges (eigenvalue greater than 1) as a result of an exploratory factor analysis (EFA) including all items belonging to the constructs, or if a dominant (first) factor structure that explains more than 50% of the explained variance is detected, the CMB is considered to be at a critical level. The Harman test, which assumes that CMB has an equal effect on all research constructs, is the most frequently used statistical test against the CMB threat in the literature (61). In this study, a result of EFA, the dominant first factor's explained variance was found as 20.55 %. Therefore, it can be concluded that there is no CMB. CMB was also tested with correlation matrix procedure. According to correlation matrix procedure, a very high correlation (>0.90) between research variables is thought to be a sign of the CMB effect (62). In this study, the correlations between variables range between 0.34 and 0.46. Therefore, common method bias does not appear to be a serious concern. However, Harman's single-factor test has known limitations in detecting common method variance, and the results should be interpreted with caution. In addition to the Harman single-factor test, the Unmeasured Latent Method Construct (ULMC) method proposed by Williams et al. (63) was used with AMOS to assess the possibility of common method variance (CMB). In this method, the theoretical measurement model was first tested. Then, a second model was constructed in which all observed variables were loaded onto a common latent method factor, and the two models were compared. After adding the method factor, the mean variance explained by the method factor was determined to be 19%. This rate is below the 25% threshold considered critical in the literature. This result indicates that common method variance did not significantly affect the study results.

A convenience sampling approach was employed based on voluntary participation. All participants were invited to complete the online survey via email, and the survey was administered through Google Forms for accessibility. Participants were required to be working full-time as accountants and to be over 18 years of age. Ethical approval was obtained from the relevant committee before conducting the study (Approval No: 2024/08, dated October 31, 2024). Additionally, informed consent was obtained from all participants prior to their participation.

The questionnaire consisted of four parts. In the first part, participants were asked questions to determine their socio-demographic characteristics. The second, third, and final sections had questions about “Workplace Violence Scale” (WV), “Job Satisfaction Scale” (JS), and “Beck Depression Inventory” (BDI), respectively.

WV was developed by Chen et al. in 2004 and adapted into Turkish by Tutan and Kökalan (64). Turkish version of WV was used in this research. The scale consists of nine questions and three sub-dimensions called “Verbal Violence,” “Physical Violence” and “Sexual Violence.” An example item for verbal violence is: “In the past 6 months, I have been verbally insulted by a colleague or superior.” An example item for physical violence is: “In the last 12 months, I have been subjected to physical violence that did not result in bodily harm.” An example item for sexual violence is: “In the last 12 months, I have been subjected to rape or attempted rape.” Items were rated on a 4-point Likert scale (1 = Never, 4 = Very frequently).

The second part of the scale includes the “Job Satisfaction” scale. The scale was developed by Brayfield and Rothe (65), shortened by Judge et al. (66), and adapted into Turkish by Başol and Çömlekçi in 2020. Scale statements were measured with a 5-point Likert-type scale, which was expressed as “Strongly Disagree,” “Disagree,” “Neutral,” “Agree,” and “Strongly Agree.” The scale includes statements such as “I find happiness in my job” and “I do my job with love” (65–67).

Levels of depression among accountants were assessed using BDI, currently accepted as the principal self-report measure for the identification of depression (68). The BDI is a 21-item, multiple-choice questionnaire set developed to measure the emotional, cognitive, and physical symptoms of the subject. There are four possible responses to each question, ranging from 0 to 3, with higher scores reflecting more serious depressive symptoms. It is a measure that can be used with good reliability for individuals aged 13 years and above. Items are rated using a 4-point Likert scale, from “Not at all” (0) to “Severely” (3). The overall score, obtained by summing all items, ranges from 0 to 63. BDI scores were grouped as follows: minimal or no depression (0–13), mild depression (14–19), moderate depression (20–28), and severe depression (29–63). Participants who scored in the moderate to severe categories were considered as having elevated depressive symptoms rather than clinical diagnosis. For this tool, the Cronbach alpha score was 0.890.

All demographic variables in the analysis consisted of gender, education level, age, and job experience, and those were the control variables.

To ensure internal consistency, Cronbach's alpha coefficients of all the measurement tools used in the study were examined. The WV had a Cronbach's alpha of 0.84, while the JS had an alpha of 0.85. The BDI showed very strong reliability, with a Cronbach's alpha of 0.89. All values exceeded the acceptable threshold of 0.70, indicating high internal consistency for each scale used in the analysis.

In total, 450 accountants were invited to answer the questionnaire and 325 completed questionnaires were returned; that is a response rate of 72.2%. According to a 95% confidence interval and a 5.5% margin of error, it was estimated that a sample size of at least 318 was needed. Toward the completion of the data collection process, 325 valid responses were collected from the contributors.

All returned questionnaires were complete and usable. Participants were required to be actively employed as accountants; no further exclusion criteria were applied. The study therefore used a convenience sampling approach based on voluntary participation.

Demographic information about the participants is given in Table 1.

**Table 1:** Demographical characteristics of the participants.

Variable / Category	Frequency	Percent (%)	Valid percent (%)	Cumulative percent (%)
Gender				
Female	134	41.2	41.2	41.2
Male	191	58.8	58.8	100.0
Total	325	100.0	100.0	
Marital status				
Single	144	44.3	44.3	44.3
Married	181	55.7	55.7	100.0
Total	325	100.0	100.0	
Age				
30 and below	113	34.8	34.8	34.8
31–40	98	30.2	30.2	64.9
41–50	76	23.4	23.4	88.3
51 and above	38	11.7	11.7	100.0
Total	325	100.0	100.0	
Education level				
Associate degree	62	19.1	19.1	19.1
Bachelor's degree	221	68.0	68.0	87.1
Graduate	42	12.9	12.9	100.0
Total	325	100.0	100.0	
Work experience				
Less than 1 year	21	6.5	6.5	6.5
1–5 years	87	26.8	26.8	33.2
6–10 years	56	17.2	17.2	50.5
More than 10 years	161	49.5	49.5	100.0
Total	325	100.0	100.0	

The sample in Table 1 comprises 191 men (58.8%) and 134 women (41.2%). 55.7% of the participants are married. The age distribution indicates that a majority (65%) of the participants are 40 years old or younger, while only 11.7% are over 50 years old. In educational background, most participants have a bachelor's degree (68%), while 12.9% had graduate-level education, indicating that the sample is highly educated. Nearly half of the participants have over 10 years of work experience (49.5%), suggesting that the sample includes a large proportion of experienced professionals. Overall, the sample is distributed with respect to gender, marital status, education, and work experience, making it representative for a wide range of accountants.

### 3.3 Results

#### 3.3.1 Descriptive statistics and correlations

Descriptive statistics and correlations for variables were calculated and are presented in Table 2. The normality of each variable was first examined, and all variables were found to be normally distributed.

**Table 2:** The descriptive statistics of the data.

Variables	Average	Std. Dev	WV	JS	BDI
1. Workplace violence (WV)	2.30	1.102	1		
2. Job satisfaction (JS)	3.39	0.933	−0.342^**^	1	
3. Depression (BDI)	10.34	8.853	0.460^**^	−0.407^**^	1
4. Gender	1.59	0.493	−0.070	0.046	−0.191^**^
5. Marital status	1.56	0.498	−0.169^**^	0.009	−0.140^*^
6. Age	2.12	1.019	−0.174^**^	0.060	−0.114^*^
7. Education	1.94	0.563	−0.038	0.075	−0.050
8. Work experience	3.10	1.007	−0.106	0.067	−0.081

*N* = 325.

^*^p < 0.05, ^**^p < 0.01.

The mean and standard deviation values of workplace violence, job satisfaction, and depression were 2.30 ± 1.10, 3.39 ± 0.93, and 10.34 ± 8.85, respectively. Correlation analysis revealed a significant negative relationship between workplace violence and job satisfaction (*r* = −0.342, *p* < 0.01) and between job satisfaction and depression (*r* = −0.407, *p* < 0.01). A significant positive relationship was found between workplace violence and depression (*r* =0.460, *p* < 0.01).

WV, JS, and BDI levels were compared by gender and marital status using independent samples *t*-tests. Female employees reported higher exposure to workplace violence than male employees (*t* = 2.988, *p* < 0.001) and higher levels of depression (*t* = 3.348, *p* < 0.001). No significant gender differences were observed in job satisfaction. When comparing marital status, single employees reported higher exposure to workplace violence than married employees (*t* = 2.969, *p* < 0.001) and higher levels of depression (*t* = 2.536, *p* < 0.05).

One-way ANOVA was conducted to examine differences in workplace violence, job satisfaction, and depression across age, education level, and work experience groups. The results indicated no significant differences across these variables (*p* > 0.05). However, correlation analysis revealed a small but significant negative relationship between age and depression (*r* = −0.114, *p* < 0.05).

#### 3.3.2 Hypotheses testing

**Figure 1:**
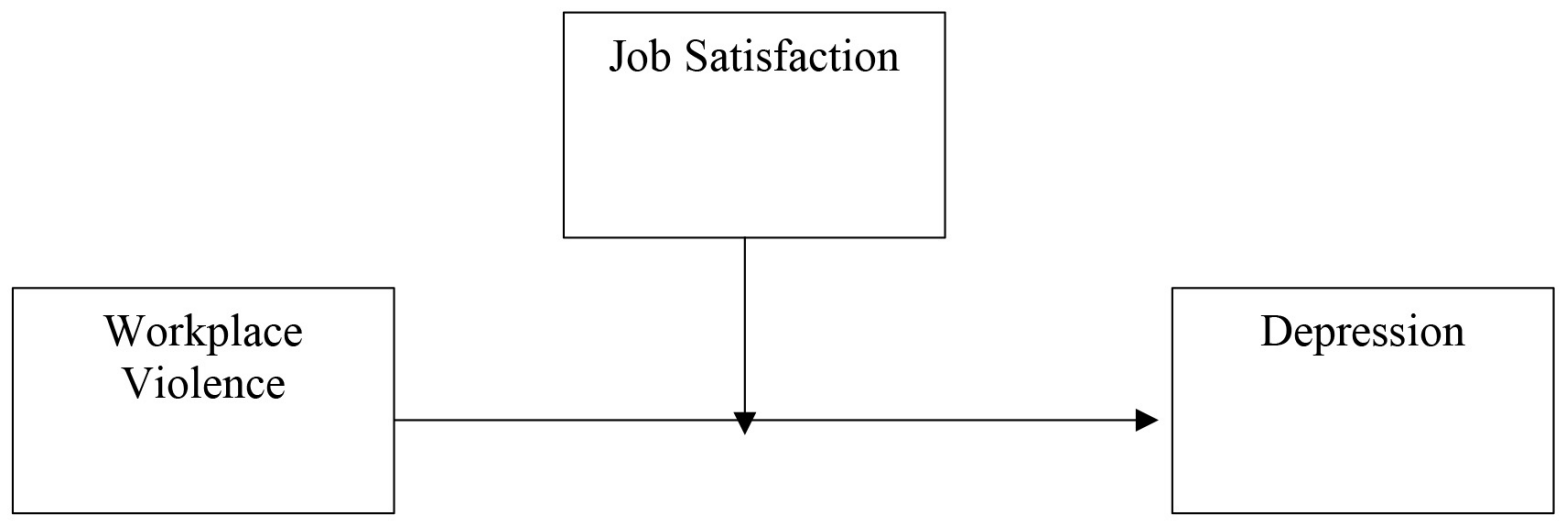
Theoretical model.

Moderator variable analysis was done with SPSS and AMOS programs. In the first step, WV, JS, and BDI variables were standardized. Then, the other independent variable (intersection variable) was calculated by multiplying the WV and JS variables. With this data, the analysis was conducted using structural equation modeling in AMOS. The theoretical model is given in Figure 1. Figure 1 presents the theoretical model, which examines the relationship between workplace violence and depression, as well as the moderating effect of job satisfaction. The model suggests that workplace violence is positively associated with the level of depression (H1), job satisfaction is negatively related to depression (H2), and job satisfaction weakens the effect of workplace violence on depression (H3). Job satisfaction, as a moderator variable, indicates that at higher levels, the relationship between workplace violence and depression is weaker.

It was feasible to comprehend the patterns of relationships between all the constructs after the parametric test and correlation outcomes. The AMOS 25.0 program (IBM SPSS AMOS 25.0, IBM Corp., Armonk, NY, USA) was used to assess structural models prior to hypothesis testing. The model demonstrated an acceptable fit to the data (χ^2^/df = 3.812; CFI = 0.911; AGFI = 0.846; GFI = 0.893; RMSEA = 0.064). Measurement models use basic criteria such as discriminant validity, composite reliability (CR) and convergent validity to assess the validity and reliability of a construct. In this study, the factor loadings of all items were above 0.5. This means that the items measuring a construct are highly related to the relevant construct and, therefore, can be regarded as valid (69). Convergent validity is expressed as the extent to which the indicators of a construct are related to each other. All AVE values greater than 0.5 mean that the constructs explain at least 50% of the variance in the observed variables. This would suggest that the latent variables are well represented by the indicators (68). Discriminant validity basically means a construct differs from other constructs and that the measures represent different concepts. The fact that the MSV for all constructs is less than the AVE values suggests that each construct is more strongly related to its own indicators and is distinct from the other constructs. This is in line with the assertion made by Hair et al. (70). Reliability of the measurement model was checked through Cronbach's alpha and CR values. Both values being above 0.7 indicate that internal consistency is high and the model is reliable. CR in particular is considered a stronger reliability measure than Cronbach's alpha value because it takes into account factor loadings (70). In this respect, it was seen that all structures met the validity and reliability criteria and it was concluded that the measurement model was strong. Although all factor loadings exceeded the minimum acceptable threshold, some items exhibited relatively lower loadings, suggesting a weaker representation of the underlying construct. However, considering that the overall reliability and validity indices (e.g., CR and AVE) were within acceptable ranges, these items were retained to preserve the theoretical integrity of the constructs. A summary of these statistical values is given in Table 3.

**Table 3:** Discriminant validity, composite reliability, and convergent validity.

Construct	Items	Factor loading	AVE	MSV	CR	C. Alpha
Workplace violence	WV1S	0.563	0.58	0.46	0.84	0.84
	WV2S	0.519				
	WV3S	0.590				
	WV4S	0.509				
	WV5S	0.626				
	WV6S	0.618				
	WV7S	0.507				
	WV8S	0.507				
	WV9S	0.556				
Depression	BDI1S	0.572	0.54	0.45	0.88	0.89
	BDI2S	0.532				
	BDI3S	0.573				
	BDI4S	0.562				
	BDI5S	0.536				
	BDI6S	0.559				
	BDI7S	0.636				
	BDI8S	0.507				
	BDI9S	0.515				
	BDI10S	0.518				
	BDI11S	0.514				
	BDI12S	0.642				
	BDI13S	0.657				
	BDI14S	0.506				
	BDI15S	0.643				
	BDI16S	0.583				
	BDI17S	0.585				
	BDI18S	0.505				
	BDI19S	0.530				
	BDI20S	0.533				
	BDI21S	0.547				
Job satisfaction	JS1S	0.572	0.51	0.48	0.85	0.85
	JS2S	0.532				
	JS3S	0.573				
	JS4S	0.562				
	JS5S	0.536				

Moderation analysis results are given in Table 4.

**Table 4:** Moderating analysis.

Path / Relationship	Coefficient	Std. error	LLCI	ULCI	*t* statistics	*p*-value	Description
WV → BDI	0.360	0.052	0.265	0.471	7.041	< 0.01	H1 accepted
JS → BDI	−0.311	0.048	−0.406	−0.216	−6.493	< 0.01	H2 accepted
WV^*^JS → BDI	−0.245	0.040	−0.315	−0.175	−6.125	< 0.01	H3 accepted

**Figure 2:**
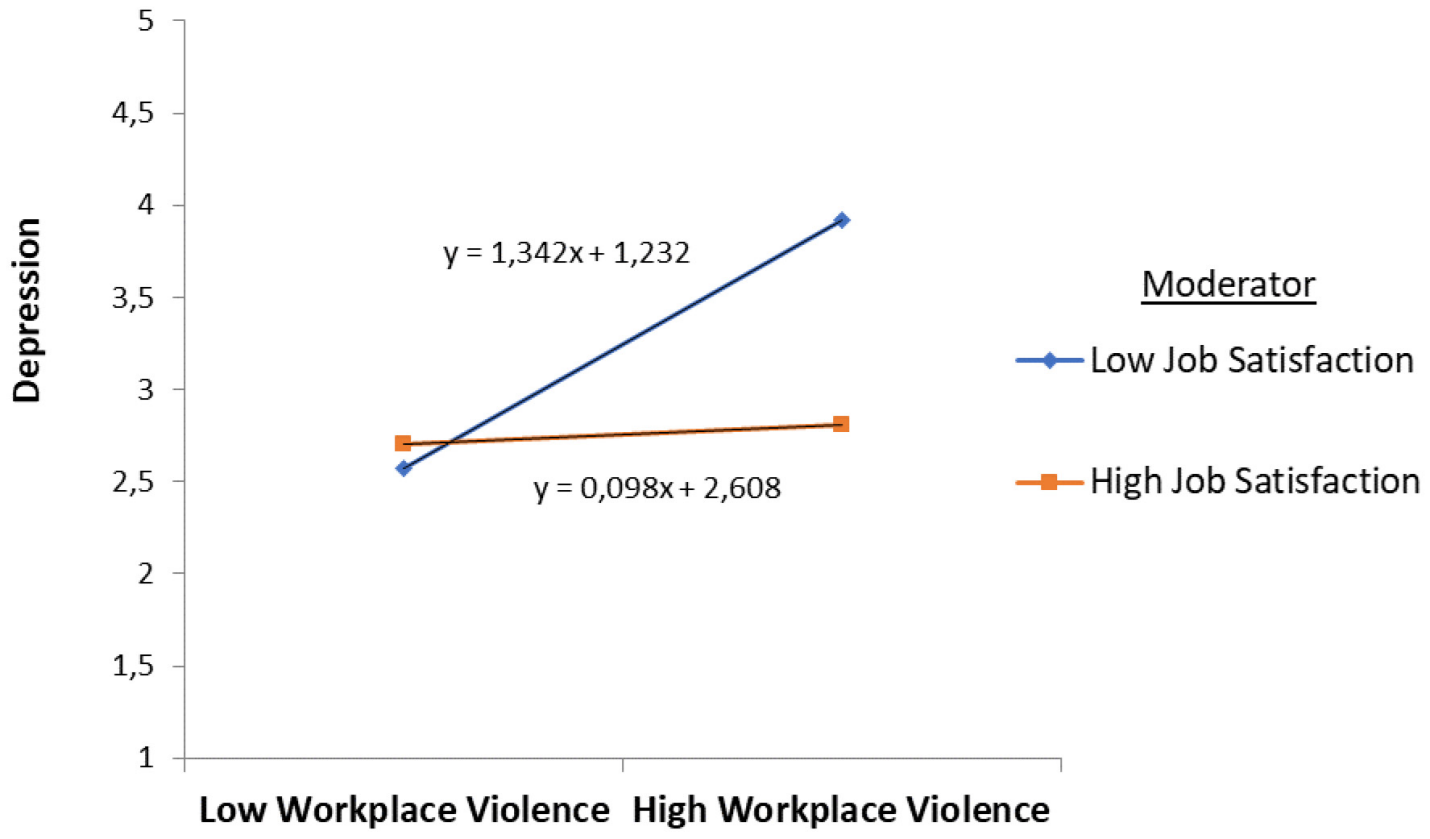
The interaction between WV and BDI.

Table 4 presents the results. Workplace violence is positively associated with depression (β = 0.360, *t* = 7.041, SE = 0.052, *p* < 0.01), supporting H1. Job satisfaction is negatively associated with depression (β = −0.311, *t* = −6.493, SE = 0.048, *p* < 0.01), supporting H2. H3 proposes that job satisfaction (JS) moderates the relationship between workplace violence and depression. The interaction term (WV × JS) is significant (β = −0.245, *t* = −6.125, SE = 0.040, *p* < 0.01), supporting H3. This finding indicates that job satisfaction appears to buffer the association between workplace violence and depression. This pattern is illustrated in Figure 2.

Before creating the interaction term, workplace violence and job satisfaction scores were mean-centered to reduce multicollinearity. The interaction term (WV × JS) was then included in the SEM model. A simple slope analysis (±1 SD) indicated that the positive association between workplace violence and depression was stronger at low levels of job satisfaction (−1 SD: β = 0.36, *p* < 0.01) and weaker at high levels of job satisfaction (+1 SD: β =0.12, *p* < 0.05).

To ensure the stability of our findings, several additional analyses were conducted. First, collinearity diagnostics indicated that all variance inflation factor (VIF) values were below 2.5, suggesting no multicollinearity problems. Second, inspection of residual plots showed that the assumptions of normality and homoscedasticity were reasonably met. These diagnostic checks support the robustness of our moderation analysis.

Figure 2 shows the moderating impact of job satisfaction on the relationship between workplace violence and depression. The graph shows that higher levels of workplace violence are associated with higher levels of depression among individuals with low job satisfaction (blue line). In contrast, for individuals with high job satisfaction (red line), the level of depression remains relatively low even at higher levels of workplace violence.

This pattern suggests that job satisfaction may function as a buffering moderator, weakening the relationship between workplace violence and psychological distress. In other words, higher levels of job satisfaction appear to attenuate the association between workplace violence and depression.

## 4 Interpretation of findings and discussion

### 4.1 Discussion

The findings of this study indicate that workplace violence is positively associated with depression among accountants, while job satisfaction appears to function as a buffering factor, with a weaker relationship observed between workplace violence and depression at higher levels of job satisfaction. These results are consistent with prior research showing that workplace violence is associated with poorer mental health outcomes. For example, (71) and Rasool et al. (2) reported that exposure to workplace aggression or violence is significantly associated with higher levels of depression and emotional distress (1, 2).

The present findings can be interpreted through an integrated perspective that combines stress-related and relational mechanisms. Workplace violence may be viewed as a psychosocial demand associated with increased emotional strain, while job satisfaction reflects how employees evaluate their work environment and available organizational support. Accordingly, employee wellbeing appears to be shaped not only by exposure to stressors, but also by how these stressors are interpreted within the organizational context.

The findings are also consistent with research in the accounting profession highlighting the negative effects of job stress and burnout on psychological wellbeing and job satisfaction. Previous studies have shown that burnout is associated with lower job satisfaction and wellbeing, while job satisfaction is linked to important outcomes such as performance and turnover intentions (8–10). In this regard, the results of this study imply that the characteristics of workplace violence could differ depending on the occupation under consideration. In the case of accounting occupations, because of their hierarchy, high performance expectations, and time pressure, workplace violence can be experienced in a more covert way, which is rooted in psychology.

As compared to previous studies on accounting occupations, the main difference between this work lies in the conclusion that workplace violence could be a potential psychosocial hazard in such jobs. Another interesting result in this study was that job satisfaction could be an effective buffer for dealing with workplace violence in accounting occupations.

This relationship supports the principles of Stress–Strain Theory, according to which stressful experiences, like workplace violence, lead to emotional exhaustion and psychological strain. According to Karasek (49) and Lazarus and Folkman (48), the lack of enough personal or organizational resources may enhance this effect. Thus, the results indicate that the process of strain is not homogenous, as it depends on the amount of available contextual resources.

Finally, regarding the influence of job satisfaction on the outcomes of psychological strain experienced due to witnessing workplace violence, the findings support the assumptions of Social Exchange Theory, since it stresses the importance of factors such as fairness and trust in relation to the reactions of employees.

Overall, the results contribute to the literature by providing evidence for the association between job satisfaction and employees' psychological wellbeing, and by showing that job satisfaction appears to reduce the strength of the association between workplace violence and depression. At the same time, job satisfaction does not eliminate this relationship as it is just connected with decreasing it.

Furthermore, according to demographic conclusions, unmarried employees and female employees might be even more susceptible to the effects of workplace violence.

The results obtained during this research do match those offered by literature sources, but at the same time, the main innovation in this field relates to the consideration of the problem of workplace violence in the accounting profession.

Furthermore, this pattern highlights the importance of considering demographic characteristics when addressing workplace violence and employee wellbeing. Companies can take advantage of interventions that focus on job satisfaction, as well as minimizing workplace violence especially among vulnerable groups. Workplace violence can be viewed as a context-related issue influenced by both individual perspectives and organizational contexts.

### 4.2 Limitations

Some limitations of the current study have to be mentioned. First, the possibility of the influence of common method bias has to be taken into account because all variables were measured by means of self-reports. Despite the application of some procedural solutions such as gathering data in several waves, this limitation is not fully overcome and might be addressed in future studies by the use of multi-source data or even more separation in time between the examined variables.

Second, while the time-lagged design was used, causal conclusions could not be made because of the absence of experiments. Thus, longitudinal designs or experiments would help reveal causal associations among the studied variables. Finally, one should note that the results obtained when comparing different groups in terms of gender and marital status have to be treated rather as exploratory findings.

Furthermore, the data were collected via self-reported questionnaire, thus there may have been social desirability or recall bias. Further studies could include some objective measures or even evaluations from a third party to make sure that the validity of the findings is not jeopardized.

Fourth, only accountants working in Türkiye were included as the sample group for this research, which might pose limitations to the generalizability of the results. Moreover, convenience sampling was used in collecting the data; therefore, the external validity of the findings might not be guaranteed as the participants volunteered to participate in the study. Other factors, such as cultural and occupational aspects of how workplace violence is perceived and experienced and the effects on mental wellbeing, should also be considered to increase the validity of the findings. Future research is recommended in other countries as well.

In addition to that, while some control variables were tested for their impacts on the relationships, other factors such as personality or organizational culture were disregarded. It would definitely make sense to incorporate these into the model in further research.

### 4.3 Future research directions

The current study offers valuable information but there are still many areas that warrant future research. For instance, future research might seek to investigate the possibility of common method variance. Researchers may choose to employ multivariate measures, or separate in time the measuring of variables to prevent bias that might result from employing one survey method.

Future research is also recommended using longitudinal designs to assess the dynamic relationship between work-related violence, job satisfaction, and depression. Moreover, intervention studies will also shed light on the efficacy of organizational interventions designed to lessen the link between work-related violence and employee mental wellbeing.

Future research can be conducted to confirm these results in other occupations and areas, like healthcare, education, or even law enforcement, to uncover risk factors that are unique to those occupations.

Furthermore, conducting additional research and using additional variables, such as emotional intelligence, coping strategies, or organizational support perception, might help reveal more information about employees' reactions to violence at work. Finally, conducting qualitative studies might help explore the experiences behind the numbers discovered during quantitative research.

## 5 Conclusion

Workplace violence is today recognized as a public health concern because of its serious implications for the psychological wellbeing of the staff members. There is adequate documentation available with regard to workplace violence statistics, and it has been noted that an increased exposure to workplace violence leads to a higher chance of developing depression. In the majority of cases, intervention strategies at the organizational level, such as zero tolerance policies, conflict resolution training, and mental health programs, have been employed.

The current research explores the link between workplace violence and the mental health of employees, with specific attention paid to the moderating effect of job satisfaction. If certain interventions are introduced by an organization, then job satisfaction can serve as a protective factor against psychological problems induced by the experience of aggression in the workplace. Our paper contributes to the scientific literature by introducing a dual-theory framework, identifying an occupational group underresearched in terms of aggression at work, and offering some practical implications. Therefore, building up a positive work environment can help to ensure employees' mental wellbeing.

In the literature, it is acknowledged that workplace violence is considered one of the variables connected to the presence of depressive symptoms in employees. While there is little evidence available regarding accountants specifically, they generally tend to work under conditions of extreme stress due to the deadline pressure, financial accountability, and long working hours. Thus, they appear to be exposed to violence and intimidation. Thus, there is a clear need for more specialized research on how workplace violence is associated with outcomes among accountants and how sector-specific interventions may help mitigate such risks.

Thus, psychosocially safe workplaces are likely to be associated with better mental health outcomes and to promote job satisfaction and performance. Workers who feel that their safety is threatened or that they have been let down by a lack of support are more likely to become disengaged, have poor morale, and be less productive. In this light, organizations should place priority emphasis on building anti-violence policies, enhancing employee support programs, and proactively encouraging trust and wellbeing through leadership.

The accounting profession is marked by heavy workloads and demanding reporting periods (8–14). “Busy-period support programs,” such as flexible working hours, counseling services, and other initiatives ahead of tax-filing deadlines, are effective in enhancing psychological resilience among employees during these periods. Organizing regular mentoring and peer-support groups fosters the mechanisms of reciprocity and social support emphasized by Social Exchange Theory, which minimize the risk of burnout (52, 58). Anti-violence and ethical communication training (7) in combination with workload rotation at the end of reporting periods may be associated with lower levels of workplace violence and depressive symptoms.

## Data Availability

The raw data supporting the conclusions of this article will be made available by the authors, without undue reservation.
